# Supplementary material for: FinDonor 10 000 study: a cohort to identify iron depletion and factors affecting it in Finnish blood donors
Source: Vox Sang. 2019 Oct 28;115(1):36–46. doi: 10.1111/vox.12856 (PMC7004091; doi:10.1111/vox.12856)
Supplement: Supplementary file 1 — Table S1 Health and lifestyle questionnaire of FinDonor 10 000 study. Table S2 Reagents and devices used for CRP, ferritin and sTfR measurements. Table S3 Counts of study donation attempts during which study blood sample was given. Figure S1 Age and Hb distributions of study participants. Figure S2 Counts of donations of study participants during study period. Figure S3 Relatedness of 760 FinnDonor participants and selected 1000 Genome reference populations. Figure S4 Ferritin versus CRP divided by BMI groups. [file VOX-115-36-s001.pdf]

FinDonor 10 000 study: A cohort to identify iron depletion and factors affecting it in Finnish blood donors

Short title: Finnish blood donor cohort

Lobier, Muriel

Niittymäki, Pia

Nikiforow, Nina

Palokangas, Elina

Larjo, Antti

Mattila, Pirkko

Castrén, Johanna

Partanen, Jukka*

Arvas, Mikko*

Finnish Red Cross Blood Service, Research and Development. Kivihaantie 7, 00310 Helsinki, Finland.

*address correspondence to: mikko.arvas@veripalvelu.fi or jukka.partanen@veripalvelu.fi

Supplementary Table 1 is provided as a separate file.

	18th May 2015 to 31st May 2016			1st June 2016 onwards		
	reagent	device	reagent manufacturer	reagent	device	reagent manufacturer
CRP	CRPL3	Roche Modular	Roche Diagnostics	CRP VARIO	Abbott Architect	Abbott
Ferritin	FERR4	Roche Modular	Roche Diagnostics	Abbott Architect Ferritin	Abbott Architect	Abbott
sTFR	Tina-quant Soluble Transferrin Receptor	Roche Modular	Roche Diagnostics	Tina-quant Soluble Transferrin Receptor	Abbott Architect	Roche Diagnostics

Supplementary Table 2. Reagents and devices used for CRP, ferritin and sTfR measurements.

Gender	Count of study donation attempts	Count
Women	1	433
Women	2	407
Women	3	316
Women	4	196
Women	5	124
Women	6	65
Women	7	22
Women	8	6
Men	1	175
Men	2	194
Men	3	175
Men	4	134
Men	5	121
Men	6	80
Men	7	60
Men	8	35
Men	9	20
Men	10	12
Men	11	5
Men	12	4
	Count of donors	2584

Supplementary Table 3 Counts of study donation attempts during which study blood sample was given.

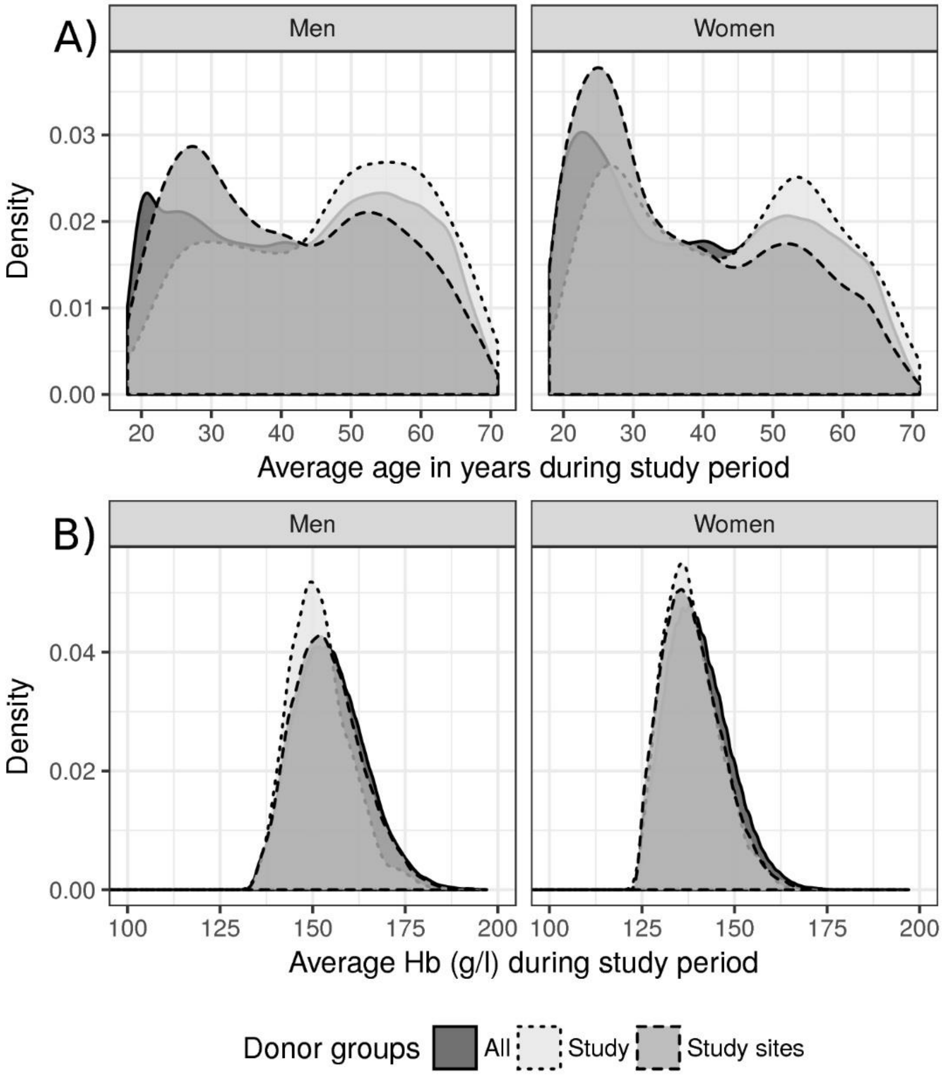

Supplementary Figure 1: Age and Hb distributions of study participants. Density function of A) age distributions of all donors (“All”), all donors that donated in the collection sites where study took place (“Study sites”) and study participants (“Study”) during the study period. Respectively the density distribution of B) hemoglobin. Average over all donation events during the study period is used as the value of age and hemoglobin. Mean of hemoglobin was “All”: 140 women and 154 men; “Study sites” 138 and 154 and “Study”: 138 and 151.

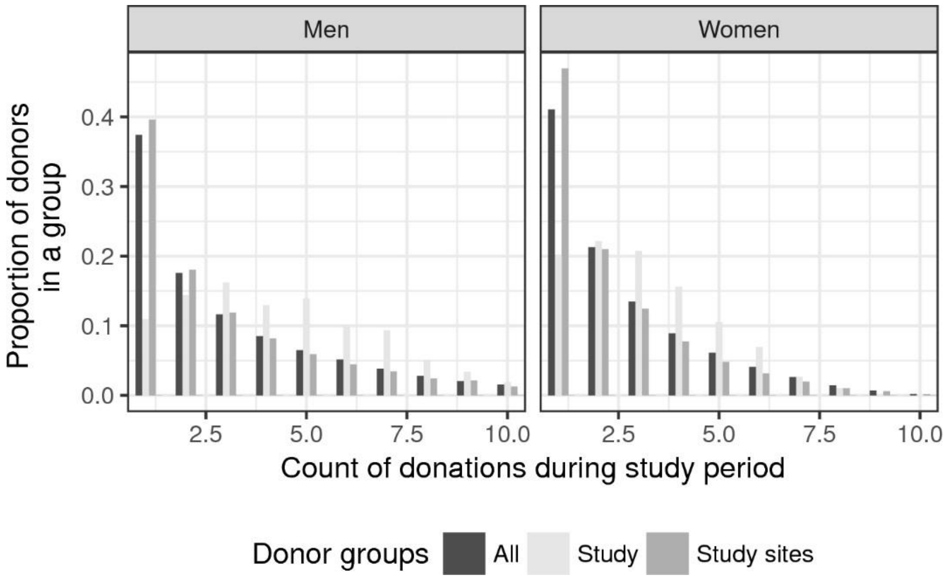

Supplementary Figure 2: **Counts of donations of study participants during study period.** “All” are donations by all donors that donated during the study at any FRCBS site. “Study sites” are donations of all donors in the collection sites where study took place. “Study” are study participants. Y-axis shows the proportion of the donors in the group (All, Study sites, Study) that donated a certain number of donations during the study period (on x-axis).

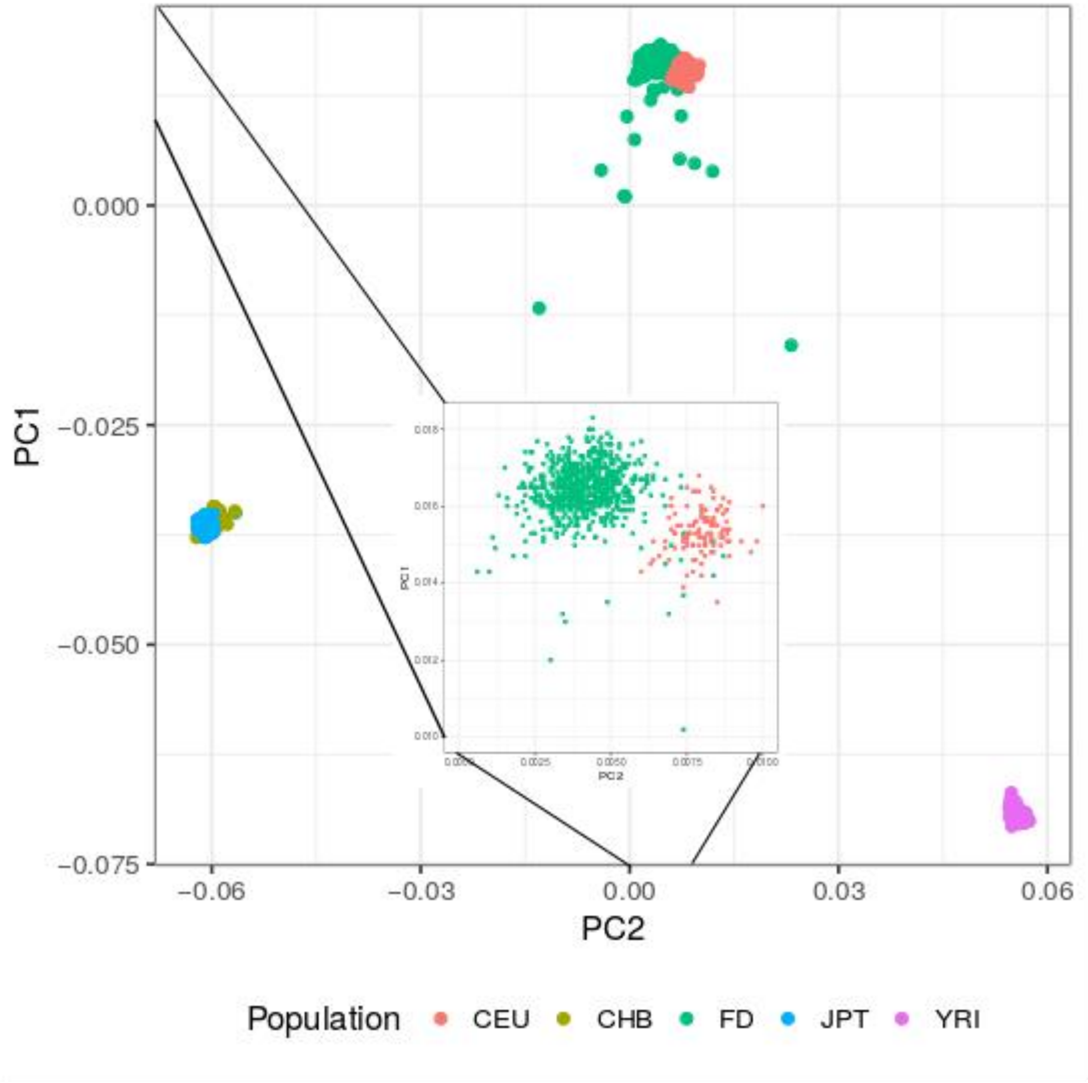

Supplementary Figure 3: Relatedness of 760 FinnDonor participants and selected 1000 Genome reference populations. Principal component analysis of similarity of exome genotyping results. Populations: CEU = Utah Residents (CEPH) with Northern and Western European Ancestry, CHB = Han Chinese in Beijing, China, FD = FinnDonor, JPT = Japanese in Tokyo, Japan, YRI = Yoruba in Ibadan, Nigeria. Inset shows the separation of FinnDonors from the CEU.

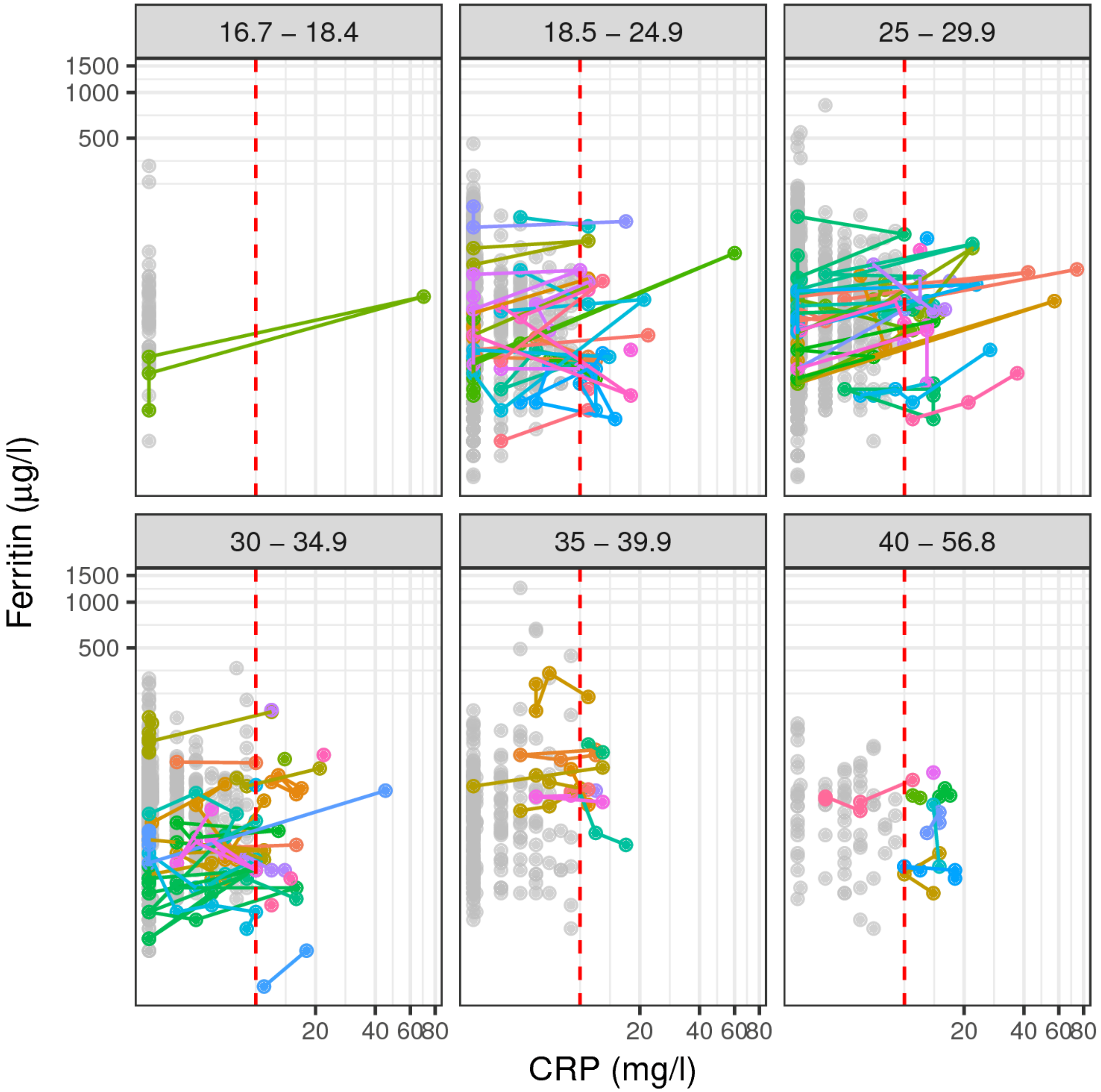

Supplementary Figure 4: Ferritin versus CRP divided by BMI groups. Each dot is a single blood sample. For individuals which have at least one sample with CRP > 10 mg/l (red dotted vertical line) dots are colored in the same color and connected by a line. Due to lack of distinguishable colors, colors are recycled from an individual to another. To aid visual separation of individuals the data is divided into 6 plots by WHO BMI based obesity classes.

Supplementary methods

Genotyping was performed at the FIMM Technology Centre, Helsinki, Finland. DNA samples from the Finnish cohort were extracted using the QIAamp DNA Blood Mini Kit (Qiagen, Germany). Imputation was carried out as in [1] according to instructions of [2] using plink 1.90b3.29 [3] for quality filtering and IMPUTE2 [4] for the actual imputation with 1000 Genomes Phase 3 [5] as a phased reference. Post-imputation filtering excluded variants having an IMPUTE2 INFO-field <0.5. After post-imputation filtering 11 880 443 variants were included. PCA (Principal Component Analysis) of the imputed and filtered dataset was carried out with eigensoft version 6.1.4 [6].

1. Hyvärinen K, Ritari J, Koskela S, et al. Genetic polymorphism related to monocyte-macrophage function is associated with graft-versus-host disease. *Sci Rep*. 2017;7(1):1-10. doi:10.1038/s41598-017-15915-3

2. Anderson CA, Pettersson FH, Clarke GM, Cardon LR, Morris AP, Zondervan KT. Data quality control in genetic case-control association studies. *Nat Protoc*. 2010;5(9):1564-1573. doi:10.1038/nprot.2010.116

3. Purcell S, Neale B, Todd-Brown K, et al. PLINK: A Tool Set for Whole-Genome Association and Population-Based Linkage Analyses. *Am J Hum Genet*. 2007;81(3):559-575. doi:10.1086/519795

4. Howie BN, Donnelly P, Marchini J. A Flexible and Accurate Genotype Imputation Method for the Next Generation of Genome-Wide Association Studies. Schork NJ, ed. *PLoS Genet*. 2009;5(6):e1000529. doi:10.1371/journal.pgen.1000529

5. Gibbs RA, Boerwinkle E, Doddapaneni H, et al. A global reference for human genetic variation. *Nature*. 2015;526(7571):68-74. doi:10.1038/nature15393

6. Patterson N, Price AL, Reich D. Population Structure and Eigenanalysis. *PLoS Genet*. 2006;2(12):e190. doi:10.1371/journal.pgen.0020190
