# Supplementary material for: FinDonor 10 000 study: a cohort to identify iron depletion and factors affecting it in Finnish blood donors
Source: Vox Sang. 2019 Oct 28;115(1):36–46. doi: 10.1111/vox.12856 (PMC7004091; doi:10.1111/vox.12856)
Supplement: Supplementary file 2 [file VOX-115-36-s002.pdf]

FIN Donor 10,000 HEALTH AND LIFESTYLE SURVEY

Donor no.: Study ID:

General

Sex

( ) Male
( ) Female

The following questions are about how you perceive your health.

1. Have you ever been diagnosed with any of the following conditions? (Please tick all that apply):

[ ] Elevated blood pressure
[ ] Elevated cholesterol levels
[ ] Elevated blood sugar levels
[ ] None of the above
[ ] Don't know

2. Have you ever been diagnosed with a low haemoglobin (Hb) count (below 125g/l for women or below 135g/l for men)?

( ) Yes
( ) No
( ) Don't know

2.1. If yes, have you ever been diagnosed with anaemia?

( ) Yes
( ) No
( ) Don't know

Health status

3. Have you ever undergone medical tests because of a high haemoglobin count?

( ) Yes
( ) No
( ) Don't know

3.1. If yes, what was the cause of the high haemoglobin count? (Please tick all that apply):

[ ] Cause not found
[ ] Smoking

[ ] Medication I'm taking
[ ] Iron overload disorder (e.g. haemochromatosis)
[ ] Don't know
[ ] If other, please specify ____

Health status

4. During the past four weeks, have you taken anti-inflammatory pain medication (such as aspirin, ibuprofen or ketoprofen)?

( ) Yes, daily
( ) Yes, almost daily
( ) Yes, occasionally
( ) No
( ) Don't know

Health status

5. How would you rate your recent health in general?

( ) Excellent
( ) Very good
( ) Good
( ) Moderate
( ) Poor
( ) Don't know

6. How would you rate your health compared with other people of similar age?

( ) Excellent
( ) Very good
( ) Good
( ) Moderate
( ) Poor
( ) Don't know

Functional ability in daily life

7. Based on your health, how would you rate your ability to carry out the following daily activities?

	Very limited	Fairly limited	Slightly limited	Not limited at all	Don't know
A. Light chores, such as moving a table, vacuuming or cycling.	( )	( )	( )	( )	( )
B. Climbing	( )	( )	( )	( )	( )

several flights of stairs.

8. During the past four weeks, have your physical symptoms (such as pain) or health in general interfered with your work or other daily activities?

( ) Yes
( ) No
( ) Don't know

9. During the past four weeks, has your mental health interfered with your work or other daily activities?

( ) Yes
( ) No
( ) Don't know

10. Have you felt that you were able to accomplish less than you would have liked to?

( ) Yes
( ) No
( ) Don't know

10.1. If yes, what are the activities that you were unable to carry out as normal, or less able than you would have liked, because of your health? (Please specify below)

____
____
____
____

Mental state

11. During the past four weeks, how often have you felt...

	all of the time	most of the time	much of the time	some of the time	a little of the time	Not at all	Don't know
A. calm and relaxed?	( )	( )	( )	( )	( )	( )	( )
B. full of energy?	( )	( )	( )	( )	( )	( )	( )
C. sad?	( )	( )	( )	( )	( )	( )	( )

12. During the past four weeks, how often has your physical or emotional health interfered with your social activities (such as visiting friends or relatives)?

( ) All of the time
( ) Most of the time
( ) Much of the time
( ) Some of the time
( ) A little of the time
( ) Not at all
( ) Don't know

Sleep habits

13. How many hours do you sleep on average

A. per night (round to the nearest half hour)?

____ hours

B. over a 24-hour period, including nights and daytime naps (round to the nearest half hour)?

____ hours

14. Sleep habits

	Yes, always or most of the time	Yes, occasionally	Seldom or hardly ever	No	Don't know
A. Do you think you get enough sleep?	( )	( )	( )	( )	( )
B. Do you feel tired during the day?	( )	( )	( )	( )	( )

Health-related lifestyle habits

The following questions are about your use of vitamins, dietary supplements, iron supplements and alcohol and your diet in general.

15. During the past four weeks, have you taken...

	Yes, daily	Yes, 4-6 times a week	Yes, 1-3 times a week	Less than once a week or not at all	Don’t know
A. multivitamin tablets/supplements?	( )	( )	( )	( )	( )
B. vitamin C tablets/supplements?	( )	( )	( )	( )	( )
C. iron tablets/supplements?	( )	( )	( )	( )	( )

16. Were you given iron tablets when you last donated blood?

( ) Yes; Retafer (30 tablets)
( ) Yes; HemoJern (20 tablets)
( ) Yes; don’t remember which
( ) No
( ) Don’t know

16.1. If yes, how many iron tablets did you take?

( ) All or almost all
( ) About half
( ) Less than half
( ) None
( ) Don’t know

Lifestyle/diet

17. Do you follow a special diet?

( ) No special diet
( ) Meat-free
( ) Lacto-ovo-vegetarian
( ) Vegan
( ) Don’t know
( ) If other, please specify ____

18. How often do you have...

	Several times a day	Once a day	4-6 times a week	1-3 times a week	Less than once a week	Never	Don’t know
A. red meat (beef, pork, lamb, game) as the main course?	( )	( )	( )	( )	( )	( )	( )
B. cutlets?	( )	( )	( )	( )	( )	( )	( )
C. fish?	( )	( )	( )	( )	( )	( )	( )
D. eggs?	( )	( )	( )	( )	( )	( )	( )
E. fruit and berries?	( )	( )	( )	( )	( )	( )	( )
F. salad and vegetables?	( )	( )	( )	( )	( )	( )	( )
G. fruit juices?	( )	( )	( )	( )	( )	( )	( )
H. wholemeal products (such as porridge, bread, muesli)?	( )	( )	( )	( )	( )	( )	( )

Lifestyle/ alcohol consumption

19. How often do you have...

	Several times a day	Once a day	4-6 times a week	1-3 times a week	Less than once a week	Never	Don’t know
A. milk or other dairy products?	( )	( )	( )	( )	( )	( )	( )
B. coffee?	( )	( )	( )	( )	( )	( )	( )
C. tea?	( )	( )	( )	( )	( )	( )	( )

	Never	Hardly ever	A few times a month	A few times a week	Daily or almost daily
D. beer?	( )	( )	( )	( )	( )
E. wine?	( )	( )	( )	( )	( )
F. spirits?	( )	( )	( )	( )	( )

Lifestyle/smoking

20. Do you smoke?

( ) Yes, daily
( ) Yes, occasionally
( ) No

20.1. If yes, how long have you smoked?

____ years

20.2. If no, have you smoked in the past?

( ) No, I have never smoked
( ) Yes, for ____years in total

Exercise

21. How would you rate your current physical condition?

( ) Very good
( ) Good
( ) Fairly good
( ) Moderate
( ) Fairly poor
( ) Very poor
( ) Don’t know

22. Which of the following statements best describes your typical day (work, studies, leisure)?

( ) Mainly sedentary with very little walking.
( ) My day involves walking but not lifting or carrying heavy loads.
( ) I walk and lift things regularly.
( ) I perform strenuous physical work that involves lifting or carrying heavy loads, digging, shovelling, chopping or other similar activities.
( ) Don’t know

23. On average, how much time do you spend each day doing light everyday physical activity, such as cycling, walking or rollerblading? Tick the alternative that best describes the average time in the course of a year.

( ) Less than 15 min.
( ) 15 min. – less than half an hour
( ) Half an hour – less than an hour
( ) An hour or more
( ) Don’t know

Exercise

24. How often do you exercise/do sports in your free time?

( ) Never
( ) Less than once a month
( ) 1-2 times a month
( ) About once a week
( ) 2-4 times a week
( ) More than 4 times a week
( ) Don’t know

Exercise

25. Which of the following are similar to the exercise you do in intensity (please tick all that apply)

[ ] Walking
[ ] Alternating between walking and light running (jogging)
[ ] Jogging
[ ] Fast-paced running
[ ] Don’t know

26. On average, how long do you spend each day in your free time on activities that are sedentary (such as doing handicrafts, reading, watching television, playing computer games, surfing the internet or sitting in a car)?

( ) Less than an hour
( ) An hour – less than two hours
( ) Two hours – less than four hours
( ) Four hours or more
( ) Don’t know

Weight and height

27. How much do you weigh?

____ kg

28. How tall are you?

____ cm

FOR WOMEN:

<u>Questions for women</u>

29. Do you use, or have you ever used, birth control pills, the contraceptive patch, vaginal ring or the contraceptive implant?

( ) No, never.
( ) Yes, at the moment; for ______________________________________years
( ) No but have done in the past; for
____ years

30. Do you use, or have you ever used, an intrauterine contraceptive device (coil)?

( ) No, never.
( ) Yes, at the moment; for _______________________________________years
( ) No but have done in the past; for
____ years

31. Do you use, or have you ever used, a hormonal intrauterine device?

( ) No, never.
( ) Yes, at the moment; for ______________________________________years
( ) No but have done in the past; for
____years

<u>Questions for women</u>

32. Do you still have periods?

( ) Yes, regularly
( ) Yes, irregularly
( ) No. I had my last period in ____

32.1. If yes, how long does your menstrual flow last on average? (in days)
____

Questions for women

33. How old were you when you had your first period? (age in years)
____

34. Have you given birth?

( ) No
( ) Yes, when? ____

35. If yes, did you breastfeed?

( ) No
( ) Yes; for ____ months (in total for all children)

36. Have you ever had trouble conceiving or having a baby?

( ) I don’t know, I have never tried.
( ) No
( ) Yes

37. Have you had a hysterectomy?

( ) No
( ) Yes, when? ____

Quality of life

How would you rate your quality of life?

( ) Very poor
( ) Poor
( ) Average
( ) Good
( ) Very good

Education

What is the highest level of education you have attained?

( ) Secondary education
( ) Vocational college
( ) Upper secondary education (i.e. high school, sixth form college)
( ) Post-secondary qualification (i.e. university of applied sciences, polytechnic)
( ) University; undergraduate degree (bachelor’s degree)
( ) University; graduate or post-graduate degree (master’s degree, doctoral degree)
( ) Don’t know

Employment status

Which of the following best describes your primary occupation (occupation that takes up most of your time or represents your main source of income)?

( ) Full-time employment
( ) Part-time employment (also partial retirement)
( ) Student
( ) Retired
( ) Unemployed or laid-off
( ) Stay-at-home parent or carer
( ) Other
( ) Don’t know

THANK YOU!
